# Professional Success

**Published:** 1844-02

**Authors:** 


*Professional Success—Gravity versus Hilarity.*—Our contemporary of the Gazette has favored the profession with an Essay on Professional Success, which is good in its way, though not exactly after the manner of Dean Swift, nor Rabelais, nor Goldsmith, nor even quite so light as Addison's. But *n'importe*, it inculcates some very sound decorum and morality, and, if somewhat prosy, shares that fault in common with many excellent discourses. But the point to which we intend to allude is one generally discussed by our contemporary—the advantages of seriousness. He observes:

"Seriousness, not occasional but habitual; earnestness, not affected but real, are useful in securing the confidence of the sick, and indispensable in the studies which prepare us for practice; and if the unvarying liveliness of a mercurial temperament, seems, in some instances, to have been a prime element of professional success, we may rely on it that such unwearied cheerfulness has only lightened the labors of conscientious activity; that it has been kept up by a consciousness of duties performed, not used as a substitute for duties neglected. Of those who can set the table in a roar, how many can leave it at the calls of duty?"

True as this may be, it cannot be deemed new, as their very charter designates the members of the College of Physicians "viri *tristes* et docti," and, no doubt, their solemnity stands them in stead. Who has not remarked some learned man, bending over a dubious stool, with a face in which are displayed all that seriousness and earnestness, so powerfully inculcated by our contemporary; and who, at the same time, has not noted the admiration which such philosophical devotedness to the cause of science and his patient, has excited in the attendant mother, or the aunt, or, above all, in the grandmother? The well weighed words, the Burleigh shake, of a professional opinion, as dark as the darkest Delphic oracle, and quite as well suited to whatever may turn up, are part of that gravity which must pervade the sayings and doings of a doctor, and to the uninitiated savour a little of humbug. In fact it is often, in this composite world of ours, difficult to separate the latter abused, but well used, instrument from the "conscientious earnestness" which it represents.

It must be agreed, we think, *nem. con.*, that doctors of physic should be grave, at any rate before their patients. Who, then, is to be lively? for something like fun may surely be permitted to the faculty. We fancy it must be given up to those queer fellows, the surgeons—whose business of passing catheters, tying piles, and curing claps, does not necessarily require such length of visage, or intense profundity of thought. And hence, we suppose it is, that so many of these same surgeons have been chatty, good-humored sorts of men, not indisposed to crack either a bottle or a joke, and have not greatly suffered in the estimation of the world notwithstanding. Far be it from us to defend this. We merely chronicle the fact, and at the present time of day, when to look wise is almost as good as to be so, and to scorn pleasantry is held by many as a certain evidence of mind, we would caution the rising generation against being misled by the lives of such persons as Cooper or Abernethy.—*Medico-Chirurgical Review*, October, 1843.

